# Case Report: CSF pseudocyst in the breast

**DOI:** 10.4103/0971-3026.57210

**Published:** 2009-11

**Authors:** L Dayananda, GA Karthik, DG Santhosh Kumar, Yameen

**Affiliations:** Department of Radiology, Narayana Hrudayalaya, No. 258/A, Bommasandra Industrial Area, Anekal Taluk, Bangalore - 560 099, India

**Keywords:** Cerebrospinal fluid pseudocyst, ventriculoperitoneal shunt

## Abstract

A cerebrospinal fluid (CSF) pseudocyst can rarely occur in the breast. It usually results from damage to a ventriculoperitoneal (VP) shunt during a mammogram or augmentation breast surgery. If fluid collection is seen in close proximity to the VP shunt, it should raise the suspicion of a CSF pseudocyst.

## Introduction

Cystic lesions in the breast are classified as simple, complicated, and complex. A cerebrospinal fluid (CSF) collection is an uncommon cause for a simple/complicated cyst in the breast. We report one such unusual case with classic imaging features on USG.

## Case Report

**Figure 1 (A, B):**
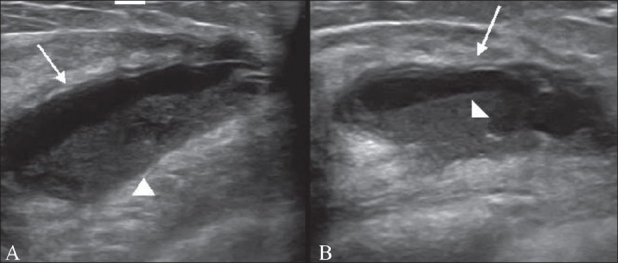
Longitudinal USG images of the medial quadrant of the breast show a cystic lesion (arrow) with debris in the dependent part (arrowhead)

**Figure 2 (A, B):**
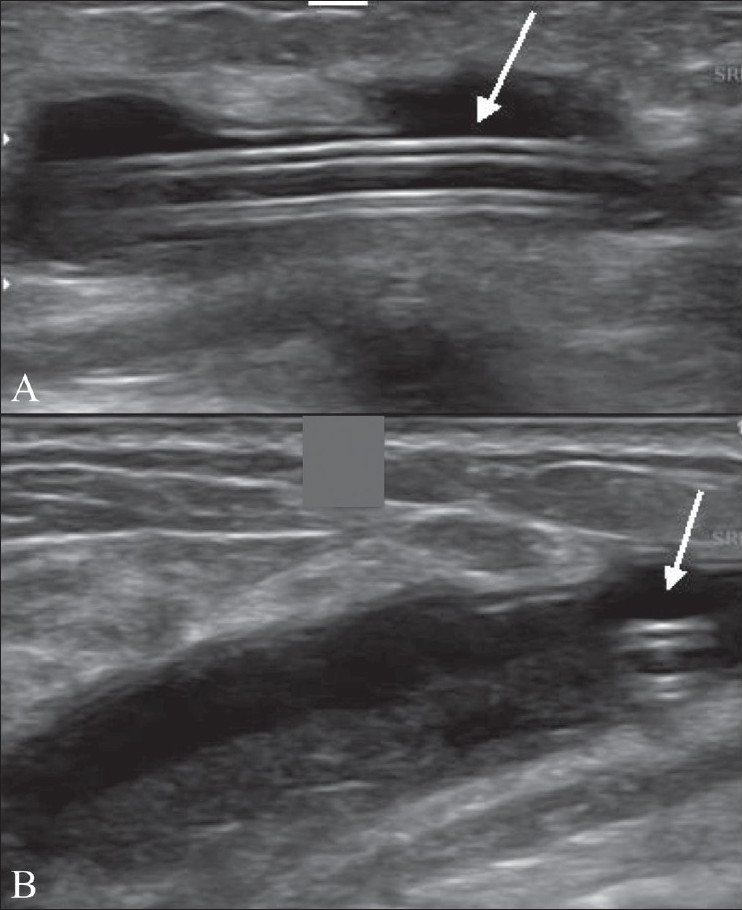
Longitudinal (A) and transverse (B) USG images show the ventriculoperitoneal shunt (arrow) in the peripheral part of the cyst

A 24-year-old female presented with a swelling in the right breast of 4 days' duration. There was no history of fever. USG showed a cystic lesion at the 9 o'clock position (i.e., in the medial quadrant). The cyst wall was very thin and debris was seen in the dependant part of the cyst [Figure 1]. A tube-like structure was seen eccentrically within the cyst [Figure 2]. On questioning, the patient revealed that she had a history of tuberculous meningitis for which she had been treated with antituberculous medication and a ventriculoperitoneal (VP) shunt. She denied any recent breast surgery, trauma, or mammography. In view of the history and the USG findings, a diagnosis of CSF pseudocyst was considered. This was confirmed at surgery, during which infected fluid was drained and the shunt tube was removed.

## Discussion

VP shunts are placed to decompress an obstructed CSF drainage pathway.[1] CSF is thus drained into the peritoneal cavity through the subcutaneously placed VP shunt. In general, the shunt is made up of a ventricular catheter, a reservoir, a valve, and a long distal catheter.[1] The distal catheter is tunneled under the skin of the scalp, neck, and chest to reach the peritoneal cavity.

Shunt dysfunction could be due to disconnection, infection, obstruction, or migration of the tube. A pseudocyst results due to leak from the drain tube, which can occur anywhere in its course. Most pseudocysts (3.2%) are seen in the abdomen.[1,2] A CSF pseudocyst can occur in the breast due to damage to the shunt tube during mammography or breast surgery. These cysts are termed pseudocysts because they lack an epithelial lining. USG is the ideal modality for investigation. Any fluid collection in close proximity to a VP shunt should raise the suspicion of a CSF pseudocyst.

The common differential diagnosis of a cystic lesion in the breast includes simple cyst, abscess, oil cyst, galactocele, fibroadenoma, intracystic papilloma, atypical ductal hyperplasia, infiltrating ductal carcinoma, and infiltrating lobular carcinoma.[3] Cysts in the breast are classified as simple, complicated, and complex. Simple cysts are those that have an imperceptible wall and contain anechoic fluid.[4,5] When the cyst contains low-level internal echoes and debris, it is called a complicated cyst. Debris, unlike a solid mural nodule, changes position within the cyst cavity with a change in the patient position. When the cyst contains thick walls, thick septa, and intracystic masses, it is called a complex cyst. Complicated cysts are further subclassified into four types: type 1 cysts have a thick outer wall or internal septa or both; type 2 cysts contain one or more intracystic masses; type 3 cysts are mixed, being predominantly cystic with solid components; type 4 cysts are predominantly solid, with cystic foci. It is important to classify the lesion on USG because simple and complicated cysts generally do not warrant biopsy or fine needle aspiration.[3,5]

## Conclusion

A CSF pseudocyst is a rare cause of breast mass. It should be considered in any patient with a history of VP shunt. USG is the modality of choice in such cases. Special care should be taken while performing mammography in a patient with a history of VP shunt because the procedure can cause tube rupture and lead to the formation of a CSF pseudocyst.
